# Supplementary material for: Loss of Argininosuccinate Synthetase-1 (ASS1) Occurs in Esophageal Adenocarcinoma and Represents a Promising Biomarker for Therapy with Pegargiminase
Source: Cancers (Basel). 2025 Nov 11;17(22):3624. doi: 10.3390/cancers17223624 (PMC12650347; doi:10.3390/cancers17223624)
Supplement: Supplementary file 1 [file cancers-17-03624-s001.zip › cancers-3972434-supplementary.pdf]

A

B

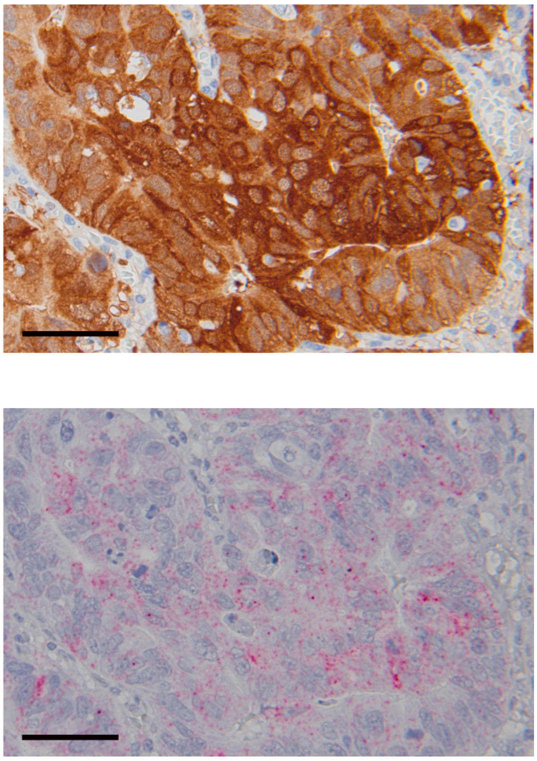

Supplementary Figure S1. Exemplary pictures of (A) immunohistochemical staining and (B) the corresponding RNAScope staining. Sidebar: 50 µm.
